# Infiltration therapy in the context of cartilage surgery

**DOI:** 10.1007/s00402-023-04964-1

**Published:** 2023-07-04

**Authors:** Wolfram Steens, Wolfgang Zinser, Philip Rößler, Thomas Heyse

**Affiliations:** 1 Department of Orthopaedics, University Medicine, 18057 Rostock, Germany; 2 Orthopaedic-Neurosurgery Center, Roentgenstrasse 10, 45661 Recklinghausen, Germany; 3 Orthoexpert, 8724 Knittelfeld, Austria; 4 AUVA-Unfallkrankenhaus Steiermark, 8775 Kalwang, Austria; 5 Joint Center, Middelrhine, 56068 Koblenz, Germany; 6 Department of Orthopaedic and Trauma Surgery, University Hospital Bonn, 53127 Bonn, Germany; 7 Center of Orthopedics and Traumatology, University Hospital Marburg, 35033 Marburg, Germany; 8 Orthomedic Joint Center, Frankfurt Offenbach, 63065 Offenbach, Germany

**Keywords:** Articular cartilage, Cartilage defects, Hyaluronic acid, Platelet-rich plasma, Adipose tissue-based cell therapy

## Abstract

Guideline-based surgical cartilage therapy for focal cartilage damage offers highly effective possibilities to sustainably reduce patients’ complaints and to prevent or at least delay the development of early osteoarthritis. In the knee joint, it has the potential to reduce almost a quarter of the arthroses requiring joint replacement caused by cartilage damage. Biologically effective injection therapies could further improve these results. Based on the currently available literature and preclinical studies, intra- and postoperative injectables may have a positive effect of platelet-rich plasma/fibrin (PRP/PRF) and hyaluronic acid (HA) on cartilage regeneration and, in the case of HA injections, also on the clinical outcome can be assumed. The role of a combination therapy with use of intra-articular corticosteroids is lacking in the absence of adequate study data and cannot be defined yet. With regard to adipose tissue-based cell therapy, the current scientific data do not yet justify any recommendation for its use. Further studies also regarding application intervals, timing and differences in different joints are required.

## Introduction

The treatment of focal cartilage damage has led to exponential development of both surgical and biologic therapies under the influence of arthroscopic surgery. Early-stage cartilage damage is clinically silent, as structural changes typically precede clinical signs and symptoms of pain. Biologic therapeutic approaches to this include platelet-rich plasma (PRP), stem cells or bone marrow aspirates (BMAC), and hyaluronic acid (HA). Surgical options are microfracture (MFx) alone or augmented [1, 2], direct repair [3, 4], autologous chondrocyte implantation (ACI) [5], autologous matrix-induced chondrogenesis (AMIC) [6], mosaicplasty [7], and osteochondral allograft transplantation [8].

Basically, a symptomatic, full-thickness, focal cartilage damage in the absence of arthritis is still the classic indication for cartilage regenerative therapy. Vogelmann et al. on a cohort of 10,000 patients in Germany calculated that under a timely guideline-compliant matrix augmented chondrocyte transplantation mACT, 21% of impending total knee arthroplasties (TKA) can be avoided [9]. In addition, a previous failed therapy of cartilage damage is a negative prognostic factor and has a lasting effect on the course of treatment [10, 11]. The strict separation of chondral and osteochondral defects is based on the recognition of the importance of the subchondral bone and the high clinical relevance of osteochondral defects, especially in younger patients [12]. In the context of postoperative treatment following cartilage surgery support can be provided with orthobiological infiltration therapies. The aim is to create optimal conditions for the regeneration of hyaline cartilage replacement tissue. Such procedures have not yet been clinically established, but are gaining in importance due to their potential to improve the quality of outcomes.

The goals can be pain relief, anti-inflammation, or support of regeneration process through accelerated or/and improved hyaline cartilage matrix synthesis. This article describes in addition to the current recommendations of the Working Group Tissue Regeneration of the German Society of Orthopedics and Trauma Surgery (DGOU) perioperative treatment options in the context of current complementary biological infiltration therapies [13].

## Hyaluronic acid (HA)

Hyaluronic acid (HA) is a high-molecular weight glycosaminoglycan naturally occurring in synovial fluid and extracellular matrix formed from chains of repeating disaccharide units. Its function is to lubricate the joint and absorb shock. Molecular fragmentation, increased synoviocyte production, and synovial fluid dilution associated with joint effusion are causes of both concentration and molecular weight decreases. HA, including cross-linked HA products, are classified according to molecular weight: (1) low (500–730 kDa); (2) intermediate (800–2000 kDa); and (3) high (2000–6000 kDa) (Table 1).

**Table 1:** Selection of commercially available hyaluronic acid preparations according to differences in molecular weight and dosage

Preparation name (manufacturer)	Ingredient	Molecular weight (kDa)	Dose
Hyalgan (Fidia Pharma)	1% Sodiumhyaluronate	500–730	20 mg weekly (5 injections)
Synvisc (Sanofi)	0.8% Hylan G-F 20	6000	16 mg weekly (3 injections)
Synvisc-One (Sanofi)	0.8% Hylan G-F 20	6000	48 mg single injection
Supartz (Bioventus)	1% Sodium hyaluronate	620–1170	10 mg weekly (5 injections)
Euflexxa (Ferring B.V.)	1% Sodium hyaluronate	2400–3600	20 mg (3 Injections)
Gel-One (Zimmer)	1% cross-linked Hyaluronate	No data	30 mg single injection
Orthovisc (DePuy Synthes)	1.5% Sodium hyaluronate	1000–2900	30 mg weekly (3–4 injections)
Monovisc (Anika/Pendopharm)	2.2% cross-linked Hyaluronate based on manufacturers cross-linking procedure	1000–2900	88 mg single injection
GenVisc 850 (Adant) (OrthogenRx)	1% Sodium hyaluronate	620–1170	25 mg weekly (5 injections)
Hymovis (Fidia Pharma)	0.8% Hexadecylamid, Hyaluronic acid- derived	500–730	24 mg weekly (2 injections)
Gelsyn-3 (Gel-Syn) (Bioventus LLC)	0.84% Sodium hyaluronate	1100	16.8 mg weekly (3 injections)
Durolane (Bioventus LLC)	Non-animal based stabilized hyaluronate	No data	60 mg single injection

Exogenous, injected hyaluronic acid stimulates synthesis of endogenous HA and may also act as a free radical scavenger [14]. HA also contributes to the inhibition of nociceptor and enzymatic cartilage degradation and stimulates the synthesis of extracellular matrix (ECM) components by synovial fibroblasts. In addition, it promotes chondroprotection by reducing the loss of proteoglycan in cartilage tissue and prevents apoptosis of chondrocytes. Furthermore, it promotes HA degradation by reducing production of pro-inflammatory cytokines and reduces the induction of pain mediators [15].

Increased molecular weight decreases the rate of enzymatic degradation and improves residence time in the joint. In addition, higher molecular weight (HMW) has greater anti-inflammatory and proteoglycan synthesis-promoting effects, as well as better maintenance of joint lubrication and viscoelasticity. HA is an intrinsic constituent of both the articular cartilage matrix as well as the synovial fluid with rheological properties, which are slowly lost in the osteoarthritis (OA) process upon the decrease of the molecular weight. Increased concentrations of free radicals, inflammatory cytokines and cleavage enzymes can also be detected in the aging cartilage matrix [16]. High concentrations of these pro-inflammatory agents lead to molecular fragmentation of HA. Therefore, with increasing age concentration of HA in synovial fluid decreases by 33–50% [17]. Another study showed more effective matrix metalloproteinase (MMP) inhibition for lower-molecular weight (LMW) products [18].

Due to the numerous mechanisms acting on the joint structure and function, HA injection for osteoarthritis is not only effective in relieving pain but also by possible disease-modifying effects. Although these developments are promising, proof of disease-modifying properties requires further clinical investigation.

Furthermore, it remains a challenge to repair osteochondral defects, which is largely due to the lack of suitable artificial or biological tissue matrices that can repair the damaged regions adequately and mechanically resilient and promote tissue regeneration. Hydrogels have emerged as a promising class of biomaterials for the regeneration of soft and hard tissues whereby in particular cell-loaded hydrogels open up new possibilities for cell therapy.

HA as a linear polysaccharide is the most abundant component in cartilage and an important aggrecan component which organizes the extracellular matrix (ECM) of the cartilage into elastic structures. The ECM of articular cartilage is a highly functional, dense connective tissue, whose restrictive barriers, however, hinder endogenous cell migration. Therefore HA-based hydrogels are one of the most promising naturally derived biomaterials for osteochondral tissue-engineering (OTE) and cartilage tissue engineering (CTE) which enables artificial cartilage constructs with high mechanical properties by promoting chondrogenic differentiation and ECM production, restoring the embryonic microenvironment and thus favoring the regeneration process [19].

Kaplan et al. reported improvement in regeneration in an animal study on partial thickness defects. A timely use of IAHA infiltrations compared to a NaCL control group showed significant differences with regard to cell viability and to proteoglycan content and cell morphology [20].

For continuous improvement of the results of IAHA injections, there is always need for more research for a better understanding of the factors contributing to cartilage regeneration.

The course of osteoarthritis varies from patient to patient and can affect the effectiveness of viscosupplementation. It is difficult to predict the type of responder because study results often do not take into account the degree of osteoarthritis. However, certain trends seem to be emerging:

1. Joint space narrowing.
  Moderate osteoarthritis is the indication of choice for intra-articular hyaluronic acid injection (IAHA), with efficacy being better in moderate joint space narrowing (Kellgren and Lawrence grades II and III) regardless of the joint. However, some studies have reported efficacy in advanced (Grade IV) knee OA, where IAHA injection can provide symptom relief pending arthroplasty [21]. Advanced hip OA, on the other hand, is not responsive to it.
2. Compartment localization
  Femorotibial osteoarthritis represents the ideal indication. In moderate femoropatellar osteoarthritis, IAHA injection appeared to be less effective, with a response rate of about 50%, according to an open British study [22].
3. Acute inflammatory arthritis
  In acute inflammation with severe joint effusion, an IAHA injection is not indicated because synovitis is associated with accelerated articular cartilage degradation and the effectiveness of HA depends less on dilution in the effusion fluid than on enzymes and oxidants (hyaluronidases, free radicals) which break down HA chains and reduce HA effectiveness. The acute episode should be treated primarily with corticosteroids.
4. Other osteoarthritis factors
  Co-morbidities (unstable meniscal tear, ligament laxity, limb deformity, osteochondral lesions, etc.) must be considered and treated in parallel with viscosupplementation as they are factors predisposing to osteoarthritis. In the case of joint-preserving surgery, an IAHA injection can be given a few weeks after surgery if the pain persists. A systematic IAHA injection at the end of the surgery cannot be recommended as it is poorly evaluated in the few published studies and only shows a very short-term benefit.
5. Chondrocalcinosis
  Chondrocalcinosis is very common in elderly patients, but does not constitute a contraindication for IAHA injection unless there is an acute inflammatory process [22].

Various randomized controlled trials have examined the effect of HA on pain and joint function. The effectiveness of HA is time dependent, with maximum pain reduction between 8 and 24th week post-injection. Studies showed that the effectiveness of hyaluronic acid in a second course of treatment for knee osteoarthritis was greater in patients with less severe radiographic changes [23]. Non-animal stabilized hyaluronic acid (NASHA) produced a significant reduction in pain and improved physical function and joint stiffness from baseline 26 weeks after a single injection [24].

IAHA injection is recommended in treatment-refractory patients after continuous or intermittent treatment with acetaminophen, NSAIDs, and slow-acting drugs [25]. It is recognized as a reliable and safe therapeutic approach for knee osteoarthritis. Adverse side effects such as pain or swelling were as common as in patients treated with placebo. Serious side effects are rare [26]. In a meta-analysis by Bellamy and colleagues, IAHA injections were compared randomly to corticosteroid injections (CSI) and placebo in knee osteoarthritis. Their analysis showed statistically significant differences between IAHA injections compared to placebo at 4 weeks and superiority of IAHA injections over CSI between 5 and 13th weeks. The HA group had a reduction in VAS score and WOMAC score at different time points during the study period compared to the other agents. The IAHA injections showed comparable efficacy to NSAIDs but with fewer side effects [17].

In another multicenter study, Adams et al. compared IAHA injections with IAHA injections/oral NSAID and with isolated oral NSAID and showed a significant difference to the baseline values in all 3 study groups after 12 weeks but no significant difference between the treatment groups. After 26 weeks, both HA groups showed a significant difference in pain and joint function compared to the NSAID group. Notably, the IAHA injections/oral NSAID group had a significant improvement in rest and nighttime pain compared to the IAHA group at 26 weeks [27].

Bannuru analyzed the effects of multiple oral medications (acetaminophen, diclofenac, ibuprofen, naproxen, celecoxib), intra-articular corticosteroids (CIS), IAHA injections, and oral and intra-articular placebo treatments in randomized controlled trials in symptomatic radiographically approved knee osteoarthritis. In this study, the most effective pain treatment was IAHA injections. In terms of function, intra-articular HA was also statistically better than intra-articular corticosteroid and intra-articular placebo injections [28]. Shang et al. published 35 patients with osteochondral lesions of the talus who had undergone an arthroscopic microfracture. 18 patients were treated postoperatively with an IAHA injection. Cartilage regeneration was assessed after 9 months using quantitative MRI. The cartilage thickness index and the AOFAS score were significantly better in the IAHA group [29].

In another study on osteochondral talus defects by Doral et al., the increase in postoperative scores after MFx in the HA-injection group (3rd/4th/5th week postoperatively) was significantly higher than in the non-injection group [30]. Görmeli et al. in a comparative study on MFx on osteochondral defects of the talus reported significantly improved clinical outcomes of patients who received PRP or HA single injections within the first 36 h postoperatively compared with a control group (NaCl infusion) [31].

Arthroscopic microdrilling with postoperative intra-articular injections of autologous peripheral blood stem cells and HA compared to a conservatively treated control group with HA injections and physiotherapy alone showed significant improvement in clinical and radiological outcome parameters in third- and fourth-degree chondral defects of the knee joint [32].

Since Thorn, in 1951, first injected hydrocortisone into the knee joint of a patient with rheumatoid arthritis, the anti-inflammatory effects of intra-articular corticosteroid compounds have been established. Corticosteroids have not only offered relief but also partial or complete remission of symptoms associated with this and other conditions. Because their action is prompt and effective in reducing inflammation, clinical use of steroids has been well established. This treatment, however, has serious side effects including altered metabolic, gross radiographic abnormalities at a time period over 12 months, loss of elasticity of articular cartilage, biochemical alterations, degeneration of tissue, microscopic and ultrastructural changes of chondrocytes and matrix [33] So far, there are no studies reporting on a combined therapy of both IAHA and intra-articular corticosteroids.

In 2016 Johal et al. in a review of 5 different guidelines (American College of Rheumatology (ACR) [34], Osteoarthritis Research Society International (OARSI) [35], European League Against Rheumatism (EULAR) [36], National Institute for Health and Care Excellence (NICE) [37], and the American Academy of Orthopaedic Surgeons (AAOS) [38]) in agreement with respect to the role of certain therapies in clinical practice lined out different recommendations on the use of intra-articular corticosteroids in comparison to IAHA with an overall clearer recommendation for the first mentioned.

## Platelet-rich plasma (PRP)

Platelet-rich plasma (PRP) has been used for dermatologic and maxillofacial conditions for more than 50 years. However, research and application of this treatment in orthopedics has increased recently. A high concentration of platelets is obtained from peripheral blood by centrifugation. The platelets are later subjected to degranulation after endogenous (e.g., calcium chloride, chitosan) or exogenous activation to release various growth factors and other active molecules (e.g., chemokines, extracellular matrix, proteins, nucleotides) to support the healing process and a reduction in inflammation [39]. Osteoarthritis-induced animal studies treated with PRP embedded in gelatin hydrogel indicated a reduction in osteoarthritis progression [40, 41]. Platelets are the smallest blood cells, are a nuclear, and contain the most extensive reservoir of factors responsible for tissue repair. Studies have detected inactive precursors of multiple growth factors (GF) in the microvesicles and exosomes of platelets [42]. The most relevant are platelet-derived growth factor (PDGF), transforming growth factor beta (TGF-beta), fibroblast growth factor (FGF), insulin-like growth factor 1 (IGF-1), connective tissue growth factor (CTGF), epidermal growth factor (EGF), and hepatocyte growth factor (HFG) [14, 43]. Various microRNAs involved in mesenchymal tissue regeneration and differentiation of MSC to chondrocytes were also detected in the microvesicles [44–47]. Therefore, in the treatment of articular cartilage lesions, the anti-inflammatory properties of platelet concentrates may be of central importance in tissue healing.

It is well known that an inflammatory response of appropriate magnitude and timing are critical for tissue repair, as the majority of mesenchymal repair occurs in the context of “controlled” inflammation. Consequently, a reduction in inflammation in synovial tissue would lead to a reduction in matrix metalloproteinases, which have cartilage matrix degrading properties [14]. The anti-inflammatory effect of PRP is due to a reduction in transactivation of nucleus factor kappa B (NF-κB), which is the critical regulator of the inflammatory process. Activated PRP has increased levels of hepatocyte growth factor (HGF) and tumor necrosis factor-α (TNF-α). These growth factors interfere with transactivation of NF-κB and are key components of the anti-inflammatory effect of PRP. Sanchez et al. evaluated the effect of intra-articular PRP infiltration and reported clinically significant pain reduction and improved function in a mid-term follow-up study of 40 patients with severe hip osteoarthritis [48].

Platelet concentrates (PRP) can be prepared in 4 different ways for clinical use [49]. Firstly, as a pure PRP with low leukocyte content (P-PRP). It is a preparation almost without leukocytes and with a low-density fibrin network after activation. It can be injected intra-articularly as a liquid solution or in the form of an activated gel. It is prepared by plasmapheresis and is, therefore, impractical for widespread clinical use. Anitua et al. have developed a procedure that involves centrifugation of the collected blood at 580 g for 8 min and separation of the plasma fractions by pipetting (EndoRet). The disadvantage here is the manual pipetting steps, which can affect the reproducibility of the final product [50]. Second, as leukocyte-rich PRP (L-PRP), also with a low-density fibrin network after activation, a greater platelet content than the pure PRP, and a higher leukocyte content. Similar to P-PRP, it can be injected intra-articularly as an activated gel or in liquid form. It can be prepared, advantageously for clinical use, by automated double centrifugation systems. Several commercial alternatives are available for this purpose (Harvest Smart-PreP; Harvest Technologies, Plymouth, MA, USA), (Biomet GPS III; Biomet Inc., Warsaw, IN, USA), (Plateltex; Prague, Czech Republic), (Regen PRP; RegenLab, Le Mont-sur-Lausanne, Switzerland).

In both P-PRP and L-PRP, platelet and fibrinogen activation occurs through various activating molecules (e.g., thrombin, CaCl_2_). Once activated, platelets release nearly 70% of their growth factors within the first 10 min. Within one hour, most of the stored growth factors have already been secreted [47]. Platelet-derived growth factors are first absorbed and then released by the fibrin network, which behaves similarly to the extracellular matrix, following a certain kinetics.

The release kinetics depends on the fibrin content, which varies depending on the individual platelet properties and the fibrin concentration and structure density induced by procoagulant enzymes in the gelation phase. This basic concept explains the duration of action of PRP after application.

Furthermore, it can be produced as pure platelet-rich fibrin (P-PRF or PRFM, Platelet-rich fibrin matrix). This is achieved first by slow centrifugation (approx. 1000 *g*) in a separation gel, which allows separation of the inactivated platelets and the fibrinogen-containing plasma from the red and white blood cells. This is followed by a second centrifugation at high speed (approx. 3500 *g*) and initiation of the coagulation cascade with (calcium chloride, CaCl_2_) to precipitate the fibrin scaffold in the formation of the malleable gel containing fibrin as a stabilizer of the “platelet clot”. The final product is a platelet-rich fibrin scaffold that is more rigid than that of conventional PRP. It has a fourfold higher platelet concentration and a low leukocyte content (Fibrinet PRFM; Platelet-Rich FibrinMatrix, Cascade Medical, Wayne, NJ, USA) [51]. This gel can be sutured or pressed into the defect site. Injection is not possible due to the highly viscous gel form. Due to the high content of fibrin, this PRP form can release growth factors over a prolonged period of up to 7 days with a high variability of kinetics. Increased release of growth factors was observed within the first day with a gradual decrease thereafter within 2 days for VEGF and PDGF and within 7 days for EGF and FGF [52]. Another option is the preparation of a leukocyte and platelet-rich fibrin (L-PRF), similar to the latter but with higher leukocyte content. As PPRF, L-PRF is a gel with high density of fibrin network and, therefore, cannot be injected, but only applied locally at the lesion site. It is prepared by simple centrifugation without the use of an anticoagulant. A commercial product of L-PRF is the Intra-Spin L-PRF (Intra-Lock Inc., Boca Raton, FL, USA).

The clinical application of PRP for the treatment of cartilage lesions and osteoarthritis currently raises more questions than answers, and the influence of a variety of variables (individual platelet properties and fibrin concentration, manufacturing methods, individual tissue response, use of homologous products for universal clinical application, etc.) that must be considered in this regard require further studies to explore the best form of PRP therapy [53].

PRP application in cartilage surgery arises from its growth-promoting properties and ability to help MSCs differentiate into cartilage and bone on the one hand, and its anti-inflammatory effects on the other.

Lee et al. investigated the potential of L-PRP as an adjunct to microfracture for cartilage defects up to 4 cm^2^ in knee osteoarthritis patients over 40 years of age [54]. L-PRP was injected in situ around the microfracture holes, following the principle of in situ activation. The 2-year results were convincing in terms of clinical scores (IKDC and Lysholm) and in the context of second-look arthroscopy after 4–6 months. These results suggest that PRP promotes healing after microfracture.

Several studies have investigated PRP augmentation in conjunction with collagen or synthetic implants. Dhollander et al. treated patellar cartilage defects by microfracturing (slow-drilling) and covered the defect site filled with L-PRP gel with a collagen I/III membrane (AMIC plus), a modification of the original AMIC technique [55]. After 24 months, they observed improvement in KOOS-score, Tegner activity scale, patellofemoral Kujala score, and VAS scale. The MOCART magnetic resonance imaging score showed incomplete repair with subchondral bone changes and intralesional osteophytes.

In the studies by Siclari et al., polyglycol and hyaluronic acid carrier materials were enriched with P-PRP and used to cover femoral and tibial defects previously treated with microfracture. Improvement was demonstrated in KOOS-score at 12 and 24 months. Biopsies from second-look arthroscopies at 18–24 months showed proportions of hyaline articular cartilage. Despite a lack of a control group, these studies demonstrate the efficacy of PRP in conjunction with support materials in single-stage treatments of chondral lesions [56, 57].

The combination of PRP with MSC is an alternative method of cartilage regenerative procedures in which the PRP can directly trigger the reparative properties of MSC in the defect site.

The basic idea is to combine the beneficial effect of platelet growth factors and the synergistic effect of BMSCs. Evidence for this has been provided several times by in vitro and in vivo studies, as well as in human in vivo studies as in the work of Saw et al. [32, 58–60]. Moreover, this procedure does not require cell manipulation and autologous resources, which promises promising reproducible and economical results as demonstrated by the study of Giannini et al. in talar osteochondral lesions [61]. In this study, BMSCs and PPRF were either mixed with porcine collagen powder or applied to a membrane of esterified hyaluronic acid derivative, placed in the defect site, and stabilized with platelet-rich fibrin gel. Improvement in AOFAS score and uniform repair tissue on MRI was observed. Second-look arthroscopies at 24 months showed similar macroscopic findings of articular cartilage. Better results were found in smaller lesions (less than 2 cm^2^) and in patients without prior surgery. A slight decrease in AOFAS score was observed between 24 and 48 months postoperatively [62]. These observations suggest that a combination of PRP and bone marrow concentrate may represent an interesting alternative to the various cartilage repair procedures.

A review of studies on the use of PRP-augmented matrices by Sermer et al. based on macroscopic, histologic, biochemical, and clinical outcome studies of 14 animal and 6 human studies with sometimes very different membranes and cell sources in different joints shows an overall situation favorable to the cartilage repair process but with limited comparability because of the large differences in PRP preparation and application [63].

Based on the current literature, PRP may be indicated as a non-operative option in early and moderate forms of osteoarthritis [21, 64–67]. Recent evidence also suggests that intra-articular administration of PRP may improve symptoms regardless of the degree of cartilage damage, but good subgroup analyses according to the Kellgren–Lawrence classification are often lacking [64, 68]. In this context, the use of PRP for grade 4 Kellgren–Lawrence lesions is currently discouraged because of insufficient data. PRP has the potential to improve knee function, possibly by reducing the inflammatory response and slowing the degenerative remodeling process of articular cartilage [69]. Better results with PRP are generally seen in male, young patients with less cartilage damage and a low body mass index (BMI).

In a review article, Laver et al. evaluated studies using PRP in the treatment of degenerative cartilage damage [70]. A total of 29 studies were included (nine prospective RCTs, four prospective comparative studies, 14 case series and two retrospective comparative studies). All RCTs reported improved symptoms in the PRP study arms at the final 12-month follow-up, seven of which reported significantly better outcomes. In general, all studies appear to show positive outcomes and clinical benefit from PRP, regardless of study design. Interestingly, there is a trend towards better outcomes in patients of younger age or early stages of osteoarthritis.

Only one study followed patients beyond 12 months (up to two years). While symptomatic improvement was seen at 12 months in this study, there was a significant decrease in functional scores at two years, albeit still greater than at baseline [71, 72].

Twenty studies used pure PRP (P-PRP), seven studies used L-PRP, and two studies did not document PRP leukocyte counts. Of the nine RCTs, eight reported improved outcomes using P-PRP and one using L-PRP.

Another meta-analysis by Chang et al. reinforces the results of Laver et al. considering that early stages of osteoarthritis benefit more from PRP injections than from hyaluronic acid injections with functionally superior results and longer duration of action (up to a year) [73].

Basic research on microfracture so far has been able to show that the use of PRP/PRF has the potential of modulation of the subchondral inflammatory reaction with migration and chondrogenic differentiation of mesenchymal progenitor cells [74]. Wong et al. observed both a significant cell migration and emigration of cells from cartilage fragments (minced cartilage) and an increase in cell viability and glucosamine expression of the cultured cells from cartilage fragments with regard to the minced cartilage defect supply [75]. Additionally, Wang et al. reported improved stimulation of mitotic activity and cartilage matrix formation of chondrogenic progenitor cells compared to mesenchymal stem cells through the use of PRP [76]. Furthermore, Jeyakumar demonstrated increased glucosamine and gene expression of anabolic markers under the influence of PRP in his study [77].

PRP-augmented matrices in connection with bone marrow stimulation have shown good data from animal studies and some human clinical studies (level II and IV). So far, however, there is no clear scientific, evidence-based improvement in results by use of PRP-augmented matrices compared to matrices without PRP.

## Adipose tissue-based cell therapy

Recently, surgical treatments for the reconstruction of both the articular cartilage and subchondral bone in osteoarthritis have been carefully analyzed to restore joint structure and function. There are, however, several associated problems, such as limitations of the available donor sources based on the required size and shape of the osteochondral autograft as well as a dedifferentiation of chondrocytes during the culturing process. To these problems, MSCs recently have attracted an increasing attention as a promising option for (osteo)chondral regeneration [78]. From adipose-derived MSCs (AD-MSCs), which have a low degree of pluripotency and also an ability to self-propagate, have a promising potential for regeneration of osteochondral defects. In numerous previous studies the ability of chondrogenic differentiation in vitro was examined, in particular using MSCs derived from bone marrow (BM-MSCs), adipose tissue (AD-MSCs) and other sources. From these stromal cells, AD-MSCs can be isolated most commonly [79]. Stromal cells of AD-MSCs for cartilage regeneration own the potential of both to differentiate into chondrocytes, are easier to isolate than other MSCs and have a very high cell proliferation rate.

Conventional methods for the transplantation of cell suspensions so far have not been successful in reconstructing osteochondral defects in animal experiments as MSC inserted into the defect did not remain at the site or did not survive [80]. Several studies investigated scaffolds made of materials such as collagen and hyaluronic acid, which support cell adhesion, proliferation, and chondrogenic differentiation [81, 82] and also support the seeding of stromal cells into osteochondral defects [83, 84].

The effectiveness of cell therapy for osteoarthritis yet has not been conclusively researched, but the secretion of anti-scarring factors (KGF, SDF1, MIP1a, MIP1b), anti-apoptosis factors (STC-1, SFRP2, TGF-β1, HGF), angiogenic factors (VEGF), and mitogenic Factors (TGF-α, TGF-β, HGF, IGF-1, FGF-2, EGF) can explain the associated natural repair mechanisms [85]. The clinical application of MSCs is strictly regulated at the present time and regulated in clinical practice in Europe but also the USA [86]. However, if AD-MSC are not expanded in vitro, but will be extracted in the operation room directly from the adipose tissue without significant manipulation and without use of collagenase (SVF), there is a strong permission from the United States Food and Drug Administration (US FDA) and the European Medicines Agency (EMA) for these therapies. In the latter case, they are then not classified as an “advanced therapy medicinal product” (ATMP) [87]. The clinical and arthroscopic second-look results after intraoperative AD-MSC injections, combined with bone marrow stimulation, revealed better results in a study by Kim compared to those with bone marrow stimulation alone in patients with varus arthrosis of the ankle joint who had undergone concomitant supramalleolar corrective osteotomy [88]. Similarly, Koh et al. also reported a significant improvement in the KOOS pain and symptom subscores after arthroscopic MFx for third- and fourth-degree femoral defects greater than 3 cm^2^ and AD-MSC-injection intraoperatively compared to a control group, while the other subscores did not differ significantly [89]. Qiao et al. in a three-arm study with microfracturing on the medial femorotibial compartment or on the patellofemoral compartment compared adjunctive postoperative NaCl injections at the 1st, 8th, 15th, and 22nd day with adjunctive HA injections at the same postoperative days and with AD-MSC injections at the 1st and 22nd postoperative day and HA injections at the 8th and 15th postoperative day [90]. A significant improvement in the WOMAC score and an improvement in the SF-36 score could be demonstrated after AD-MSC injections. In addition, MR-tomographic evidence of significant reduction of the articular cartilage defect and an increased cartilage volume during the course could be observed.

## Conclusion for practice

The efficacy of hyaluronic acid in acute traumatic cartilage lesions has been demonstrated in animal models. The clinical relevance is early treatment with hyaluronic acid in acute articular cartilage lesions to reduce or delay joint degeneration [20]. IAHA injection is also indicated for symptomatic, moderate, and effusion-free osteoarthritis. It is simple and well tolerated with proper injection technique. Functional outcomes have been improved by IAHA injections, sometimes significantly, in several comparative studies. Although the efficacy is only moderate, the response rate is high and thus allows the saving of opioid analgesics and NSAIDs with a better risk–benefit ratio and, in addition, endoprosthetic joint replacement may be delayed.

Hyaluronic acid injections have a chondroprotective effect especially in combination with intra-articular local anesthetics and cortisone injections [91]. Comparing the efficacy of PRP and HA, several authors recently have come to the same conclusion PRP being superior to hyaluronic acid therapy within a follow-up period of a year in terms of clinical scores with the same safety of application [92–97]. With regard to the different hyaluronic acid types (high molecular weight and cross-linked), this general statement does not fit as PRP is only significantly superior to the low-molecular-weight and non-cross-linked types of HA [93]. These results suggest that HA injections during and after cartilage therapy have a potentially positive effect on outcome and quality of cartilage regeneration.

The sole clinical application of PRP for the treatment of cartilage defects and osteoarthritis currently raises more questions than answers exist. The influence of a large number of variables (individual platelet properties and fibrin concentration, manufacturing methods, individual tissue response, use of homologous products for universal clinical use, etc.) that need to be taken into further studies are required to determine the best way of PRP therapy. Based on promising results from in vitro and in vivo studies, the use of use of PRP as an adjuvant in and after surgical treatment of cartilage damage and in mild arthritis of the knee joint can basically be rated as not harmful and potentially useful.

However, studies on the optimal number and timing of injections are needed since the available studies on this are inconclusive and relatively heterogeneous.

In summary, a positive effect of PRP/PRF on cartilage regeneration from preclinical studies is likely, but not yet proven in clinical outcome. For this purpose, randomized (e.g., against M-ACT) standardized studies should be carried out as required by the latest recommendations of the Working Group on Clinical Tissue Regeneration of the DGOU [98].

In recent years, single-stage cartilage regenerative therapies with mesenchymal bone marrow stromal cells (BM-MSC) as BMAC procedure or with fragmented cartilage pieces (minced cartilage procedure) moved into the focus of interest and also in the daily clinical routine and are not generally recommended so far. The recovery of a stromal vascular fraction (SVF) from adipose tissue compared to the BM-MSCs, which have so far been better investigated is becoming increasingly important. With regard to the optimal injection time, there is no general procedure recommended in the current literature [99]. Consequently, randomized controlled studies to determine the optimal timing of augmentation of surgical cartilage therapies by injections are mandatory for the future. An unrestricted, uniform or even standardized application recommendation for cell-based injection cannot be pronounced at the present time.

## Data Availability

The authors confirm that the data supporting the findings of this study are available within the article [and/or] its supplementary materials.
